# Frontotemporal Dementia as a Possible Manifestation of Primary Lateral Sclerosis: A Case Report and Literature Review

**DOI:** 10.1155/2022/8936467

**Published:** 2022-03-04

**Authors:** Kevin Qosja, Nicole M. Absar, Allen T. Yu

**Affiliations:** ^1^Renaissance School of Medicine at Stony Brook University Stony Brook, NY, USA; ^2^Department of Psychiatry and Behavioral Health, Stony Brook University, Stony Brook, NY, USA; ^3^Stony Brook Center of Excellence for Alzheimer's Disease, Stony Brook University, Stony Brook, NY, USA; ^4^Department of Surgery, Mount Sinai Hospital, New York, NY, USA

## Abstract

Primary lateral sclerosis (PLS) is currently defined as a restricted phenotype of amyotrophic lateral sclerosis (ALS), a neurodegenerative disease with upper motor neuron (UMN) symptoms that causes slowly progressive spasticity. The diagnostic criteria of this disorder currently do not include any effects on frontal executive or other cortical functioning. We report an 84-year-old woman diagnosed with six years of PLS who also had concurrent symptoms of difficulties in language, anxiety, emotional lability, and executive function. This case, as well as previously reported cases in the literature, is an example that shows the importance of more widespread consideration for PLS in patients with UMN signs and indications of frontotemporal dementia (FTD). Increased consideration for PLS would be beneficial for many patients and positively affect treatment, especially since patients live with the disorder for longer periods than ALS.

## 1. Introduction

PLS has classically been defined as a neurodegenerative disorder that is, according to the revised El Escorial criteria of 2015, a “restricted phenotype” of ALS [1]. The major findings associated with PLS are UMN symptoms causing a progressive spasticity [2]. PLS was discovered and named by Wilhelm Heinrich Erb in 1875 as a condition that primarily affects the lateral tract [3] which was corroborated by autopsies perform by Jean-Martin Charcot, particularly of a woman with spasticity and contractures [4]. This was in contrast to the condition of ALS, which was given to patients with both upper and lower motor neuron (LMN) symptoms. Since the original descriptions of the diseases, more studies have been noted to corroborate that the definition of PLS as a restricted version of ALS may be incomplete. Nevertheless, in common clinical practice, the diseases are still defined by their clinical findings. Since clinical findings can be interpreted in different ways, PLS can be confused with other diseases as will be seen in the case below. We use this case and a further review of the literature to argue for a wider consideration of PLS as a condition that should have considerations for cognitive symptoms and neuroimaging when diagnosing, and not solely determined based on the presence of UMN signs.

## 2. Case Presentation

Our patient is an 84-year-old female college graduate with a past medical history of hypothyroidism and PLS diagnosed in 2015. The patient was diagnosed with PLS after showing involuntary muscle tension and spasms of the upper and lower extremities, but no noted myoclonus or alien hand syndrome. By time of presentation, she was cared for by a home health aide as she was bedridden, unable to transfer herself, and had difficulty swallowing. The patient had extreme expressive aphasia but was able to understand simple one-stage questions and answered with few words. Insight was impaired. The patient lived with her son, who reported that the patient was communicating well until her husband's death in 2015 with symptoms of motor spasticity appearing that year as well. After neurologic investigation, an initial diagnosis of Parkinson's disease was given in 2015. The patient was treated with the dopaminergic medication Sinemet for multiple months, which was not beneficial. After the medication trial failed, a second neurologist confirmed her diagnosis as progressive lateral sclerosis that year. A noncontrast MRI of the brain performed in 2017 revealed mild cerebral atrophy with proportional ventricular dilation, as well as a few foci of supratentorial, periventricular, and subcortical nonspecific white matter hyperintensity. The patient's muscle spasticity persisted, and starting in 2019, she developed new psychiatric symptoms, including episodes of aggression, compulsive screaming, catastrophic anxiety, mood lability, a low frustration tolerance, and psychomotor agitation, more so during the sundowning hour causing circadian dysregulation. She would have anxiety attacks, depressive episodes with associated apathy, and involuntary emotional outbursts with loud screaming. These neurobehavioral symptoms indicated involvement of the patient's cognitive dysfunction of the executive domain of the frontal lobe. Patient's last thyroid function tests showed a TSH level of 16.94 and a normal free T4 level of 1.15 ng/dL, being managed with Levothyroxine. Further evaluation was limited as patient became fully aphasic at last examination, but the severe mood and emotional lability, impulsivity, and aggression with circadian dysregulation made the differential more in line with FTD than Alzheimer's disease or subcortical dementia, which are associated more with apathy than impulsivity. Likewise, language-based dementias would not explain the patient's aggressive behavior. Further evaluation was planned to formally diagnose the frontal lobe syndrome of the patient but following the last neuropsychiatric evaluation, patient underwent extensive hospitalization secondary to UTIs and aspiration pneumonia. The patient's last brain imaging was a noncontrast CT of the head that only revealed mild bilateral white matter hypodensities similar to common chronic microvascular ischemic disease. Patient passed away shortly after, secondary to bacteremia from multiple infections, after being discharged to home hospice.

## 3. Discussion

Presently, PLS is still considered a restricted phenotype of ALS. The issue is complicated as the exact disease process of these two is still not fully understood, leaving both disorders to still be mainly diagnosed clinically. Scientists in the community have been arguing that the definition of PLS should be expanded, and there is research to support that. Recent studies have tried to provide more objective measures to connect ALS and PLS as with one paper that compared the immunochemistry studies of PLS patients to ALS patients, which found similar ubiquitin immunohistochemistry findings [5]. Studies have also demonstrated an association between ALS and psychiatric disorders, with 15% of ALS patients meeting the criteria for FTD and up to 50% of ALS patients showing cognitive or behavioral decline [6]. The same distinction has yet to be made for PLS, despite patients like the one in this study having findings that are independent of their UMN symptoms. There has been some connection between PLS and frontotemporal degeneration. One retrospective case series of 181 patients with PLS from the Netherlands over a 10-year-period showed six of those patients (3.3%) had also been diagnosed with FTD [2]. However, a wider more systematic review found that 49% of patients with PLS have some degree of cognitive or behavioral abnormality [7], similar to the 50% of ALS patients with impairment noted above. Another followed 13 patients who would be longitudinally confirmed to develop PLS and showed that MRI imaging of these “pre-PLS” patients showed thinning of the right precentral gyrus, the site of the primary motor cortex, which is a clinical observation found in patients with PLS before they developed clinically obvious upper motor symptoms [8]. While this finding was not noted in the brain of the patient of this case study, it may have been present and disguised by the general cerebral atrophy. Perhaps with more awareness of this as a possible finding, it may be caught more often in the future. This case matches another in which the autopsy of an 82-year-old woman with PLS also showed marked atrophy of the precentral gyrus and microvacuolation and ubiquitin positive neuronal inclusions and dystrophic neurites in cortical layer II [5], concordant with the patient's history of progressive dementia. While our patient did not have these specific findings on earlier MRI, she developed symptoms that indicate involvement of this brain region. Nevertheless, these multiple studies support an intersection between PLS and FTD or some degradation of cortex tissue.

Other recent studies have included more systematic evaluation of PLS patients to consider findings beyond clinical observation, including one prospective imaging study of PLS patients which noted the widespread and symmetric frontotemporal degeneration in this cohort of mostly C90rf72-negative patients, providing observable evidence of frontotemporal pathology in PLS patients [9]. C90rf72 is a known genetic mutation noted in patients with ALS and hereditary spastic paraplegia (HSP), providing support that these patients have solely PLS and are not patients in early stages of ALS. Other studies evaluated other objective findings to define PLS, such as a retrospective evaluation of 75 PLS patients and 277 ALS patients with multiple screening instruments which found that the percentage of patients with ALS who had cognitive impairment was similar to that of patients with PLS, arguing that PLS be recategorized as a subtype of ALS rather than a restricted phenotype [7]. One prospective study analyzed C90rf72-negative PLS patients and ALS patients with whole-brain and region-of-interest scans and found that in PLS, the areas affected in PLS patients involve regions of the motor cortex more medial than those in ALS. Other differences include that PLS involves more cerebellar white and grey matter degeneration, spares the postcentral gyrus, and affects the body and splenium of the corpus callosum more, compared to more genu involvement in ALS [10]. These are observable differences in imaging findings that can be used alongside clinical observation to diagnose and treat patients with PLS without needing to rule out ALS which can be a considerable time investment. Overall, more consideration is being put into the possible neurocognitive and neurobehavioral findings of ALS. However, despite research showing how similar PLS is to ALS, that same consideration was slower to be attached to PLS. Fortunately, the literature is now becoming more proficient at addressing this. The patient of the case study was diagnosed with PLS six years ago but a full neuropsychiatric evaluation was not held until the patient had begun to deteriorate medically in a manner that interrupted the full diagnosis of the patient's frontotemporal involvement. Hopefully now with the progression of current research, these concerns can be addressed far earlier. It is expected that when patients are newly diagnosed with ALS, they are advised about the possible upcoming neurocognitive and behavioral symptoms (similar to FTD) and that possible treatments to slow the progression are discussed. Now with updated research, we hope patients with newly diagnosed PLS could have that same consideration.

## 4. Conclusion

The current definition of PLS does not mention frontotemporal dementia or any associated language, behavior, or executive function difficulties. Many in the neurologic community believes that the current definition of PLS as defined by the El Escorial criteria of 2015 is incomplete and is missing aspects of PLS that have been confirmed and reproduced by studies. Evidence has shown that PLS likely has more objective findings noticeable on MRI findings, and that behavioral changes and cognitive symptoms similar to FTD are far more common than originally believed, meaning that PLS should be included in more differentials in patients experiencing cognitive decline and motor symptoms. If PLS is not more widely considered, patients with UMN signs and cognitive impairments similar to ALS are at risk of being misdiagnosed because of a misconception that no noted LMN symptoms means the cognitive signs must not be related to the UMN signs. We present that it would be prudent when investigating patients with UMN symptoms and cognitive impairment to have more consideration for PLS. This would allow those patients, like the one in this case study, more time to prepare for and treat upcoming behavioral and cognitive symptoms.
